# Hollow-fibre infection model: adaptations for the culture and assessment of fastidious organisms

**DOI:** 10.1099/acmi.0.000744.v3

**Published:** 2024-06-28

**Authors:** Andrew Mead, Stefano Azzariti, Ludovic Pelligand

**Affiliations:** 1 Comparative Biomedical Sciences, The Royal Veterinary College, London, UK

**Keywords:** antimicrobial resistance, fastidious, HFIM, pharmacodynamics, pharmacokinetics

## Abstract

The hollow-fibre infection model (HFIM) is a valuable *in vitro* platform for emulating antimicrobial drug pharmacokinetic profiles. Despite its potential, standardized protocols for HFIM operation, especially concerning fastidious organisms, are lacking. This study addresses this gap by examining challenges in culturing *Pasteurella multocida* and *Actinobacillus pleuropneumoniae*, two fastidious organisms, in the HFIM. Our findings reveal effective strategies to prevent system clogging, involving multiple freeze–thaw cycles of horse blood, centrifugation and cell straining to enhance the clarity of the Mueller-Hinton fastidious medium defined by the European Committee on Antimicrobial Susceptibility Testing and Clinical and Laboratory Standards Institute. Additionally, we propose that the provision of a CO_2_ atmosphere, along with the utilization of gas-permeable tubing and gas vent filters, significantly facilitates the growth of fastidious organisms. Remarkably, both *P. multocida* and *A. pleuropneumoniae* were sustained for a period of up to 10 days under these optimized conditions. This study provides crucial insights into the modifications necessary to successfully culture fastidious organisms in the HFIM, paving the way for more accurate and representative *in vitro* models for antimicrobial drug testing. These advancements hold promise for advancing research in the field of antimicrobial pharmacokinetics and efficacy against challenging pathogens.

**Impact statement**

This study addresses a significant gap in research methodology by investigating the challenges associated with cultivating fastidious organisms, such as *Pasteurella multocida* and *Actinobacillus pleuropneumoniae*, within the hollow-fibre infection model (HFIM). Using innovative techniques such as multiple freeze–thaw cycles of horse blood, centrifugation and cell straining, we successfully prevented system clogging, enhancing the clarity of the Mueller-Hinton fastidious medium. Additionally, creating a CO_2_-rich environment and employing gas-permeable tubing with vent filters allows gas exchange probably facilitating the growth of these organisms, enabling their maintenance in the HFIM for up to 10 days. These findings not only offer a practical solution for culturing fastidious organisms in the HFIM but also hold significant implications for advancing research in antimicrobial drug testing, ultimately contributing to improved understanding and management of challenging pathogens.

## Data Summary

All data generated are presented within the article and all facets required for the method to be reproduced are presented. Details of all materials are provided in Table S1, available with the online version of this article.

## Introduction

Pharmacokinetic–pharmacodynamic (PK/PD) modelling is crucial for understanding the interplay between antibiotic concentration and bacterial killing efficacy. *In vitro* systems, categorized as static or dynamic, explore this relationship. Static systems maintain a constant antibiotic concentration, offering cost-effective experimentation and significantly contribute to fundamental PK/PD characterization [1], but lack clinical relevance regarding the absence of continuous drug concentration changes observed *in vivo* [2,4].

Dynamic systems address these limitations, allowing *in vitro* emulation of true patient PK profiles and exploration of time-dependent PK/PD features such as adaptive resistance, dose fractionation and growth delay [1]. Bi-compartmental models, exemplified by the hollow fibre infection model (HFIM), incorporate a central reservoir (CR) and extra-capillary space (ECS) [5,6], with the ECS mimicking a peripheral infection site [7,8]. The CR provides a reservoir for dosing and clearance, whilst the hollow-fibres ensure that the bacterial compartment is maintained and not similarly depleted. The HFIM has been qualified by the European Medicines Agency (EMA) to provide data to support PK/PD analyses [9]. However, no standardized guidelines are currently available regarding the HFIM. To ensure reproducibility of experiments, all the techniques and materials used in this study were implemented following a recent systematic review by Sadouki *et al*. [4].

Experimental data need to be in line with the European Committee on Antimicrobial Susceptibility Testing (EUCAST) guidelines for susceptibility testing to correctly relate with the epidemiological distributions for each bacteria. For susceptibility testing of fastidious organisms, including *Streptococcus pneumoniae, Haemophilus influenzae, Moraxella catharrhalis, Listeria monocytogenes*, *Pasteurella* spp., *Kingella kingae*, *Aerococcus* spp., *Campylobacter* spp. and others, EUCAST [and Clinical and Laboratory Standards Institute (CLSI)] recommends the use of Mueller-Hinton fastidious (MH-F) media [10,11]. The use of a defined medium for fastidious culture was first presented by Cartwright *et al*. [12] and consisted of Colombia base with eight further supplements; this formed the basis for the standardized MH-F consisting of cation-adjusted Mueller-Hinton broth (CAMHB) supplemented with 5 % lysed mechanically defibrinated horse blood and 20 mg l^−1^ β-NAD. This medium reduces factors that may influence susceptibility testing whilst still containing the haemin and NAD required for the growth of fastidious organisms. Furthermore, many fastidious organisms require a controlled atmosphere; for example, culture of *Actinobacillus pleuropneumoniae* is optimal at 5–10 % CO_2_ [13].

To date, there have been no reports of the use of EUCAST (and CLSI) defined MH-F medium in the HFIM and the methodological adaptations required to support this medium. Previous studies used a ‘modified fastidious broth’ (mFB) in the culture of *Neisseria gonorrhoeae* in the HFIM [14,17]. These studies reported successful cultures maintained for up to 168 h and system blockages were not reported. However, the use of mFB is not standardized and PD analysis of these results may not be harmonized with standard susceptibility data reported from other laboratories.

This study highlights the key challenges and core adaptations required for fastidious HFIM and reports the successful maintenance of *A. pleuropneumoniae* and *Pasteurella multocida* for an extended 10 day culture period suitable for long-term PK/PD studies.

## Theory and implementation

### Initial method and challenges

#### Initial set-up to assess perfusion

The HFIM consisted of a two-compartment polysulfone hollow-fibre cartridge (AP/510103; Alpha Plan) with a 20 kDa pore size cut-off, connected to a CR bottle (Fisher Scientific) via silicon tubing (Masterflex; Coleparmer). A Duet pump (Fibercell; New Market) maintained a constant flow of approximately 110 ml min^−1^ in the central circuit, regulated by one-way non-return valves (VWR) for unidirectional flow and CR–ECS equilibrium. Diluent (fresh medium), controlled by a pre-calibrated peristaltic pump (205S/530S; Watson-Marlow), entered and was iso-volumetrically removed to maintain a 172 ml CR volume. Gilson Pharmed tubing (Fisher Scientific) and Watson-Marlow manifold tubing (Watson-Marlow) were used for diluent and clearance, with internal diameters of 1 and 2 mm, respectively. The elimination tube, fixed at the meniscus, ensured a higher flow rate than diluent, maintaining total system volume. Low-pressure fittings (Bio-Rad) secured tubing connections.

MH-F, following EUCAST guidelines [10], consisted of CAMHB (Merck), supplemented with 20 mg l^−1^ β-NAD (Glentham Life Science) and 5 % lysed mechanically defibrinated horse blood (TCS Bioscience). The preparation involved three freeze–thaw cycles and a single centrifugation for clarity, although guidelines allow up to seven freeze–thaw cycles and a secondary centrifugation for increased clarity.

All system components were connected, except for the HF-cartridge, and autoclaved as a single unit to ensure total system sterility. The HF-cartridge was connected in the central-circuitous flow in a Class 2 microbiological safety cabinet to maintain sterility. MH-F was pumped around the system via manual palpitation of the duet pump tubing and all air was removed via the CR. As there was no dosing required, the dosing port was capped.

#### Initial results and challenges

Using EUCAST-defined MH-F in the HFIM, a novel approach not previously described, revealed challenges with this complex medium. Primarily, the MH-F components, such as remaining unlysed red blood cells (RBCs) and cellular debris, often blocked narrow-bore tubing and connections, impeding media flow. Blockages led to secondary issues such as increased system pressure, resulting in damage and leakage. This clogging would also impact on antimicrobial drug (AMD) administration and equilibration and rapidly block 0.2 µm filters that would be required for dosing sterility.

In the initial run, blockage occurred after 96 h in a low-pressure fitting between the diluent reservoir and the CR. In a subsequent run with increased freeze–thaw cycles (up to five cycles) for the lysed horse blood component, extending HFIM runtime, blockage still occurred by day 9 (between 208 and 216 h). The extended runtime following increased freeze–thaw cycles suggests that reducing the potential for remaining unlysed RBCs decreases the likelihood of HFIM system failure.

### Modifications/solutions

#### Challenge 1: preparation of fastidious medium

**Fig. 1.**
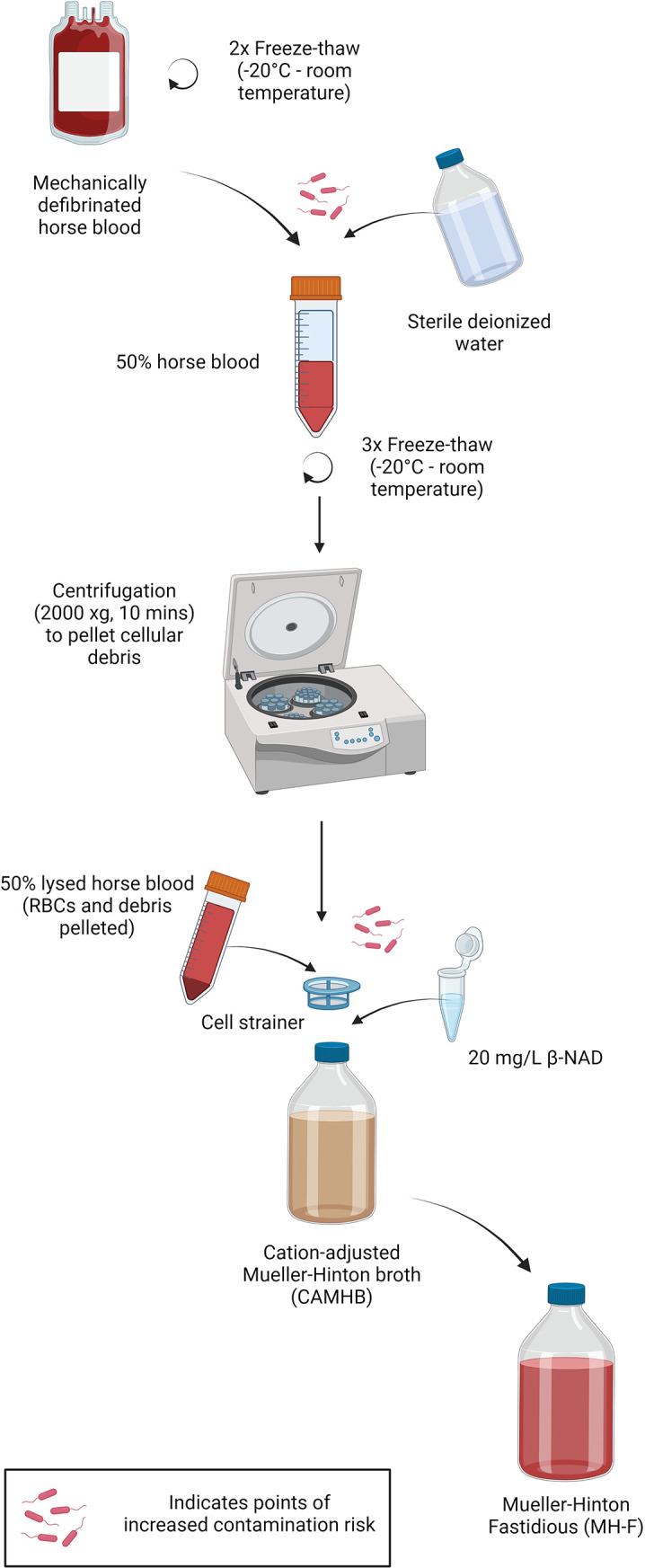
Method for production of Mueller-Hinton fastidious (MH-F) medium. Key steps for improved clarity for use in the hollow-fibre infection model (HFIM) include repeated freeze–thaw cycles, centrifugation to pellet remaining red blood cells and cell straining to remove remaining cell debris. (Created with BioRender.com.)

**Fig. 2.**
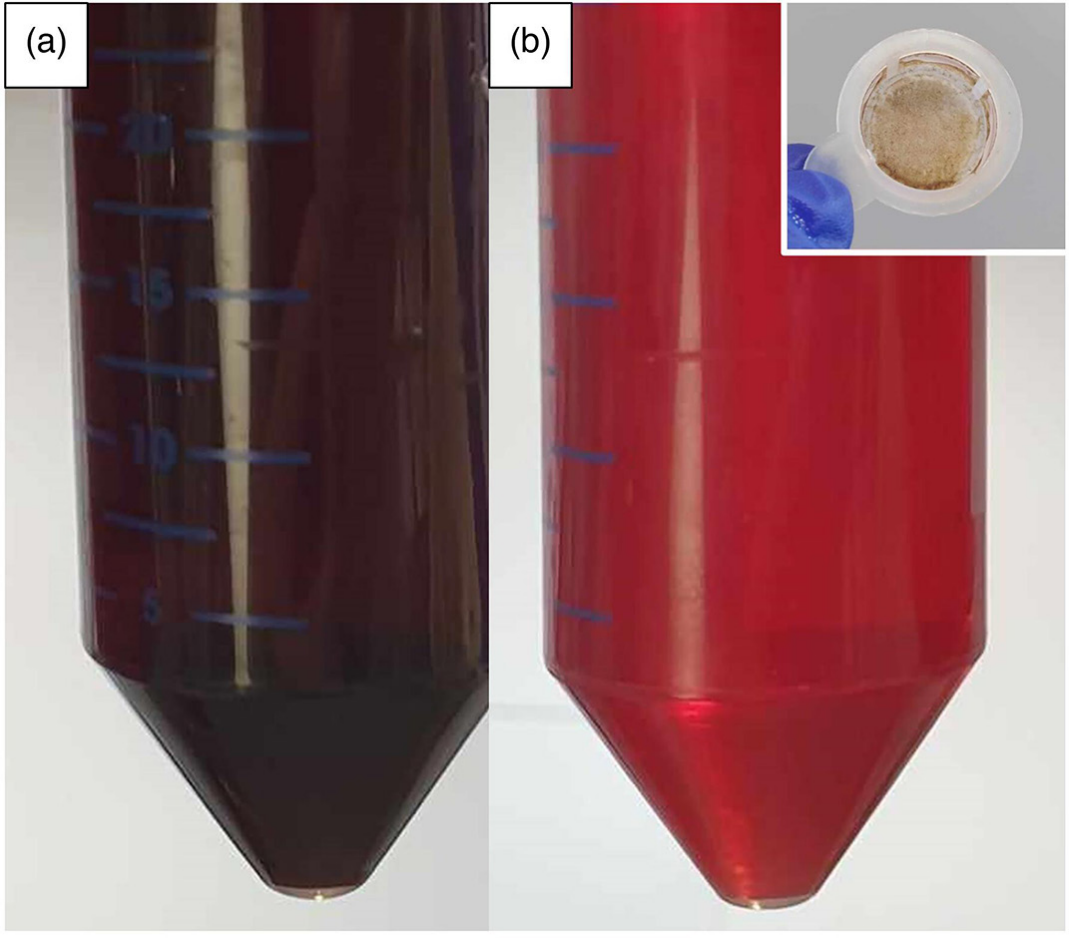
Mueller-Hinton fastidious (MH-F) medium causes clogging of narrow-bore tubing and inhibits equilibration across the hollow-fibre. Increased clarity of MH-F may be achieved through the inclusion of a cell straining stage to remove residual debris or clumps following freeze–thaw lysis and centrifugation. In this study we demonstrate five freeze–thaw cycles (−20°C to room temperature), one centrifugation (2000 g) and a single cell straining step. (a) Unfiltered and (b) filtered; inset, cell strainer after filtration.

For susceptibility testing, EUCAST recommends CAMHB or MH-F broth based on the bacterial organism. MH-F is advocated for *A. pleuropneumoniae* and *P. multocida*. However, standard MH-F preparations (as described above) lead to HFIM blockages and system failure. To address this, enhanced MH-F clarity was achieved through maximal lysis, centrifugation and an additional cell straining step. Horse blood underwent five freeze–thaw cycles, with two pre-dilution cycles and three post-dilution cycles to 50 %. Centrifugation (2 000 *g* for 10 min) removed RBCs and debris, and the supernatant was added to CAMHB through decanting with a cell strainer. This process, depicted in Fig. 1, significantly increased clarity, as evidenced in Fig. 2.

#### Challenge 2: considerations for dosing (CAMHB + filtration)

**Fig. 3.**
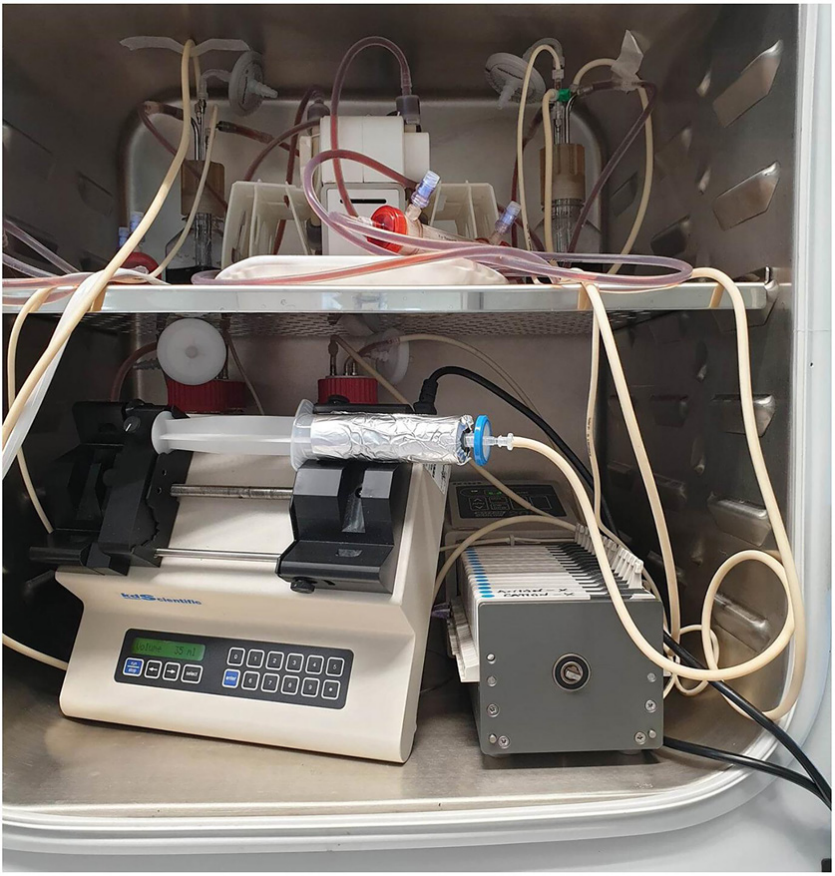
As the dosing syringe is fitted to the HFIM after sterilization it acts as an increased risk of contamination. A syringe filter reduces the risk but is rapidly clogged by MH-F. Dosing with CAMHB overcomes this challenge.

For full implementation, AMD dosing in the HFIM was achieved via bolus or infusion pump into the CR to simulate specific PK profiles. To ensure sterility, a 0.2 µm syringe filter was placed between the dosing syringe and the CR, especially if the syringe was changed during the profile. Due to the rapid clogging of this filter by MH-F, dosing was done with standard CAMHB. The dosing volume, relative to the total system volume and inflow of drug-free MH-F (diluent), was negligible and did not impact system media composition. To prevent incubator overheating, the dosing syringe was kept outside the incubator, sealed with gas exchange occurring in the CR. See Fig. 3 for an example of a dosing syringe with an associated filter.

#### Challenge 3: CO_2_ atmosphere and gas permeability

**Fig. 4.**
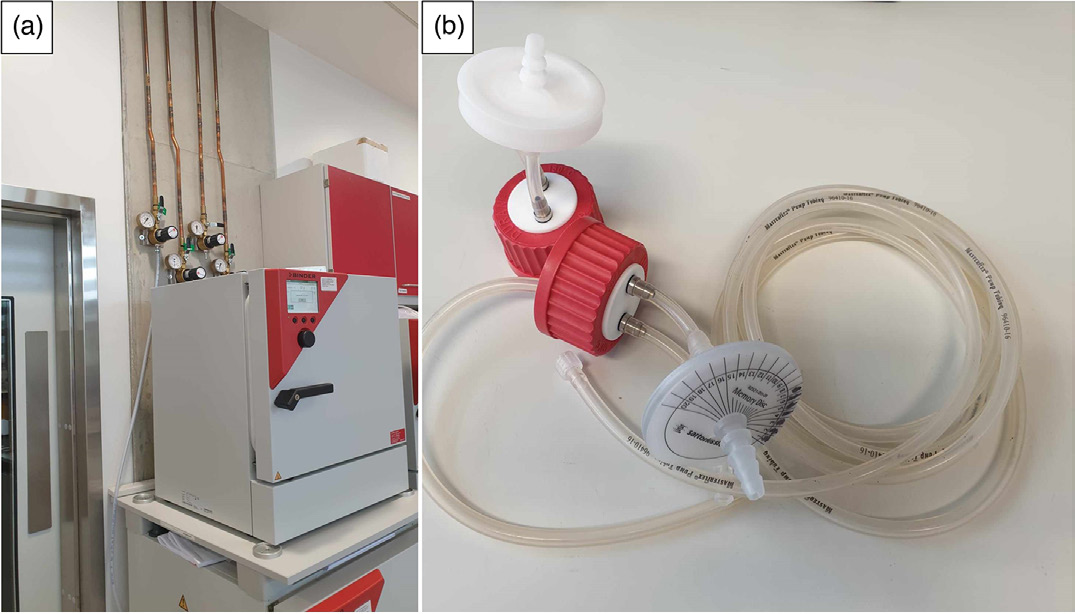
Growth of fastidious organisms requires a controlled 5% CO_2_ atmosphere. CO_2_ incubator with user-controlled CO_2_ atmosphere (a) and the inclusion of gas-permeable tubing and vent-filters (b) on all reservoirs ensure active gas exchange across the HFIM.

To maintain optimal growth cultures, including those in the HFIM, bacteria were incubated at 37 °C and 5 % CO_2_. The entire HFIM, i.e. hollow-fibre cartridge, CR, diluent and elimination reservoirs, was contained within the incubator. Gas exchange was facilitated through the incorporation of gas-permeable (O_2_, CO_2_, N_2_) silicon tubing (Masterflex; Coleparmer) which constituted the central circuit between the HF-cartridge and the CR. Furthermore, central, diluent and elimination reservoirs were equipped with an autoclavable polypropylene vent with a 0.2 µm hydrophobic PTFE membrane (StarLabs) on each two-port cap (VWR) as shown in Fig. 4.

After the set-up, the HFIM was perfused with MH-F overnight before inoculation of the bacterial load to ensure that gas equilibration was optimal before the start of each experiment.

#### Implementation

The HFIM was set-up as previously described with the additional modifications outlined in this paper.

#### Maintenance of culture in the fastidious HFIM for 10 days

**Fig. 5.**
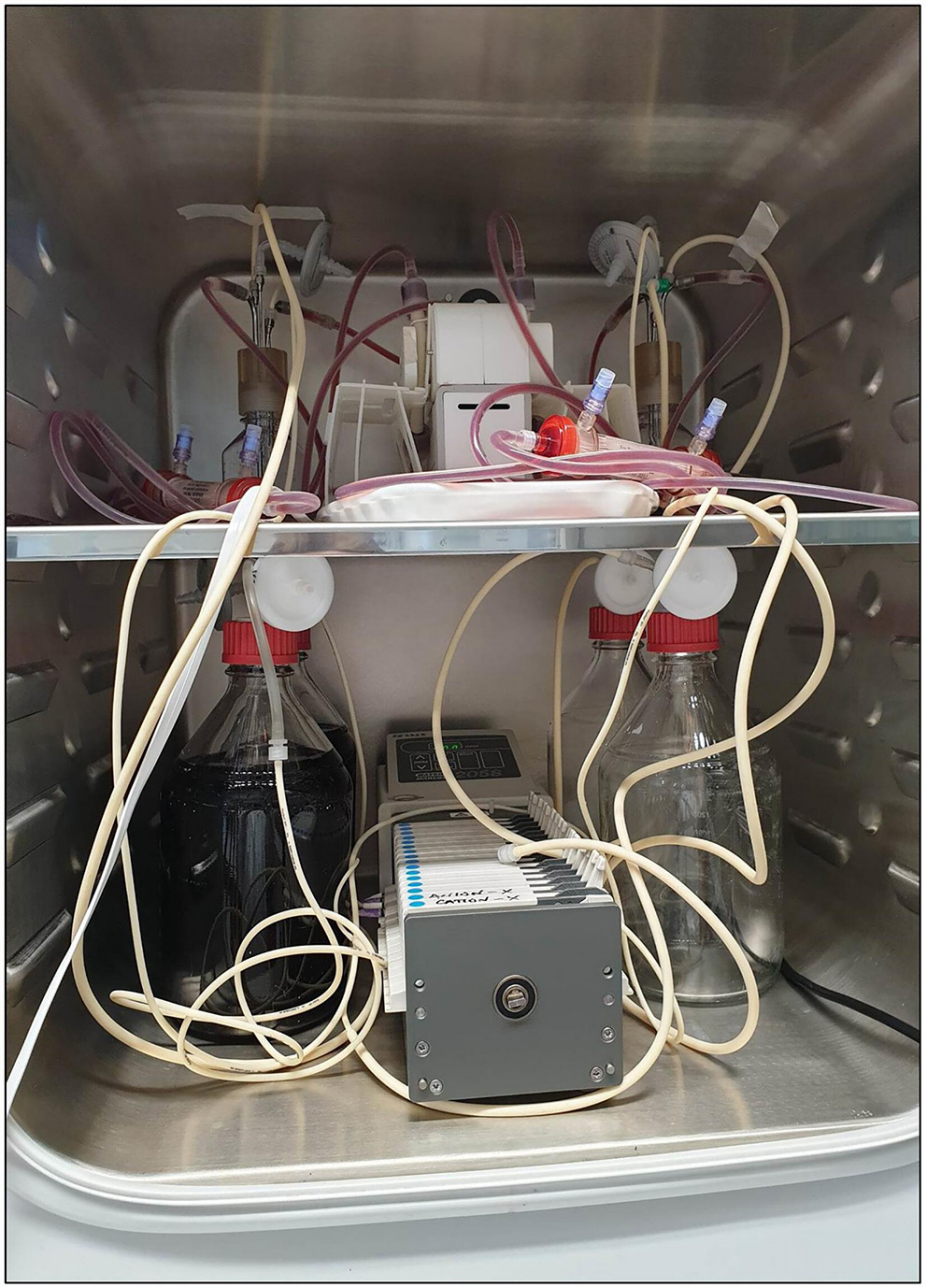
Complete HFIM set-up with Mueller-Hinton fastidious (MH-F) medium demonstrating the running of two systems using a single duet pump and single peristaltic pump. With this set-up, two simultaneous systems modelling the same PK profile and with identical or different doses can be performed in a single incubator. Drug dosing was independently controlled by a syringe pump kept either inside or outside the incubator.

Porcine respiratory isolates of *A. pleuropneumoniae* and *P. multocida* (provided by ECO Animal Health) underwent independent HFIM runs for each bacterium, with the same isolate used across studies. Inoculum preparation followed three sub-cultures on blood agar to optimize growth, adhering to CLSI guidelines (1999). Bacteria were resuspended in sterile PBS, adjusted to 0.5 McFarland standard density (approx. 1–2×10^8^ c.f.u. ml^–1^) using a densitometer (DensiCheck; bioMérieux). Dilution to a final bacterial density of approx. 5×10^5^ c.f.u. ml^−1^ occurred in 25 ml pre-warmed MH-F (or CAMHB for growth comparison) sufficient to fill the ECS of the HF-cartridge. Inoculation via ECS sample ports, using two sterile syringes, ensured homogeneous mixing. The entire HFIM was incubated at 37 °C with 5 % CO_2_, as detailed in Fig. 5.

One millilitre samples were aseptically collected from the ECS at 0, 1, 2, 4, 8, 12, 24, 32 and 48 h, then every 24 h up to 240 h of incubation. Ten-fold serial dilutions were performed in PBS and 50 µl spots of each dilution were spotted onto MH-F agar plates, which were incubated statically overnight at 37 °C and 5 % CO_2_. Tips were replaced between each dilution to avoid overestimation of the colony count caused by bacterial clumping or carry-over on the pipette tip.

After incubation, cell colonies were counted at the lowest dilution with approximately 1–50 c.f.u. and counts expressed in c.f.u. ml^−1^. The limit-of-quantification (LOQ) of the enumeration method was 20 c.f.u. ml^−1^.

**Fig. 6.**
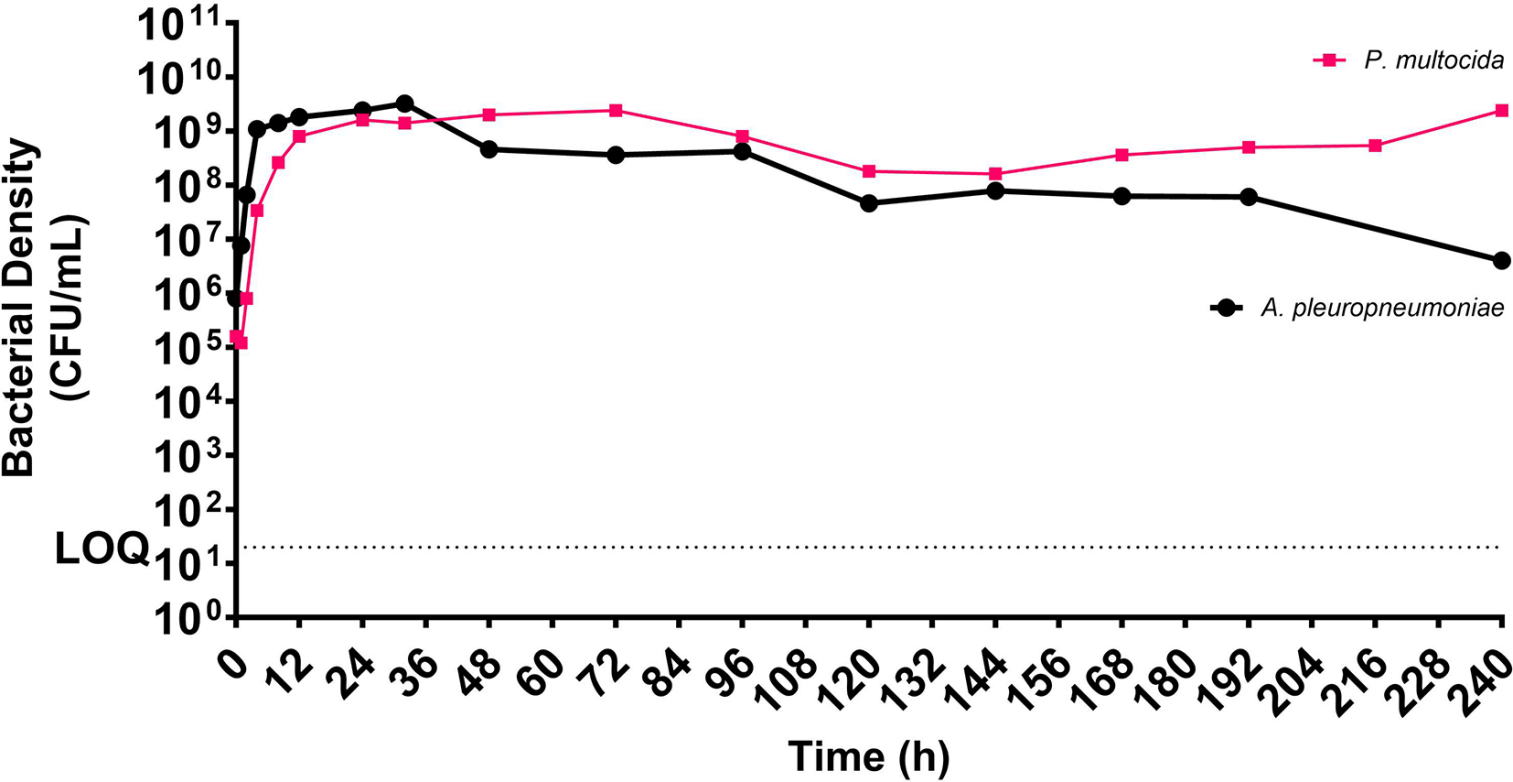
Bacterial density (c.f.u. ml^−1^; LOQ = 20 c.f.u. ml^−1^) of *A. pleuropneumoniae* and *P. multocida* cultured in the hollow fibre infection model (HFIM) in Mueller-Hinton fastidious (MH-F) medium over an extended period of 240 h (10 days).

The fastidious HFIM’s assessment for maintaining *A. pleuropneumoniae* and *P. multocida* spanned 10 days (240 h). Both species exhibited logarithmic growth without a noticeable lag phase, reaching a maximum bacterial density of approximately 10^9^ c.f.u. ml^−1^. *A. pleuropneumoniae* reached 1.08×10^9^ c.f.u. ml^−1^ at 4 h (mean doubling time=23 min), and *P. multocida* reached 0.8×10^9^ c.f.u. ml^−1^ at 12 h (mean doubling time=58.56 min). The HFIM sustained viable bacterial populations for the entire 240 h duration. However, *A. pleuropneumoniae* displayed a gradual decline from 48 h, ending at 4×10^6^ c.f.u. ml^−1^ at 240 h. *P. multocida* experienced a similar effect between 96 and 144 h, briefly dropping to 1.6×10^8^ c.f.u. ml^−1^ and regrowing to 2.4×10^9^ c.f.u. ml^−1^ (*n*=1; Fig. 6).

**Fig. 7.**
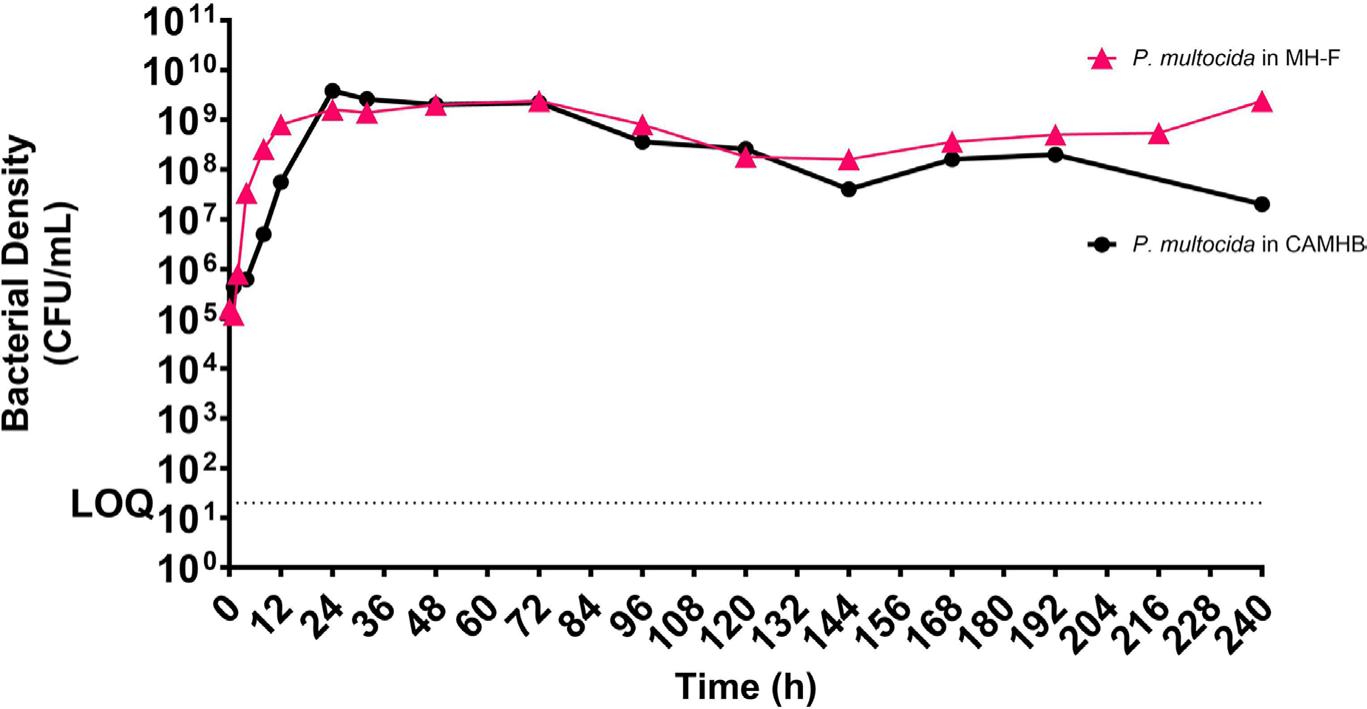
Bacterial density (c.f.u. ml^−1^; LOQ = 20 CFU/mL) of *P. multocida* cultured in the hollow-fibre infection model (HFIM) in either cation-adjusted Mueller-Hinton broth (CAMHB) or Mueller-Hinton fastidious medium (MH-F) showing a decreased growth rate over the initial 24 h in CAMHB and highlighting the importance of MH-F for fastidious organisms.

Furthermore, a parallel explorative study showed that although *P. multocida* can grow in both CAMHB and MH-F, comparison of growth in these two matrices, within the HFIM, demonstrates the importance of utilizing fastidious media. Bacterial density (c.f.u. ml^−1^) of *P. multocida* indicated that CAMHB supported a more limited, slower, growth rate than MH-F and that maintenance at the maximal carrying capacity (approx. 10^9^ c.f.u. ml^−1^) is improved in MH-F with a continual decline seen in CAMHB over 240 h (*n*=1; Fig. 7).

#### Dose validation in the fastidious HFIM

To assess if the fastidious HFIM can emulate a PK profile, a florfenicol profile in pigs was simulated to mimic a two-injection formulation based on PK data from Embrechts *et al*. [18] and Voorspoels *et al*. [19]. The HFIM was set-up as previously described without the addition of bacteria. A dosing syringe containing 20.7 µg ml^−1^ florfenicol provided two 2 h infusions 48 h apart. A constant diluent/elimination rate of 0.21 ml min^−1^ provided a constant AMD clearance, representative of that in pigs. One millilitre samples were aseptically collected from the ECS at 0, 2, 24, 48, 50, 72 and 120 h. Florfenicol was quantified by LCMS by Analytical Services International (St. George’s, University of London) with an LOQ of 0.001 mg l^−1^.

**Fig. 8.**
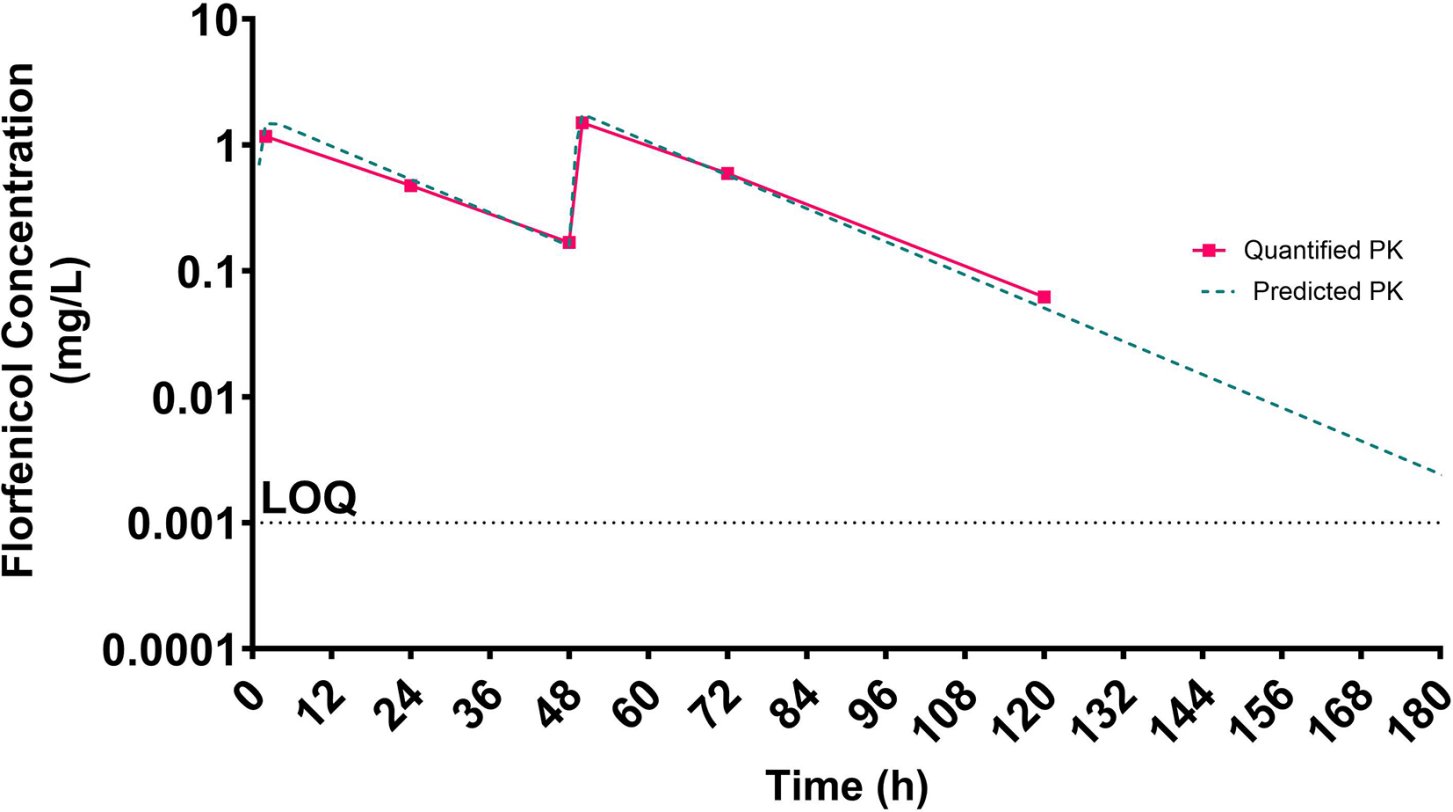
A simulated florfenicol profile representative of a two-dose administration 48 h apart. The predicted profile as defined by the PK clearance in pigs (green line) is overlayed with the LCMS quantified florfenicol concentration in the HFIM (pink line). LOQ = 0.001 mg l^–1^.

The concentration of florfenicol in the ECS of the HFIM demonstrated that the measured profile was an excellent fit of the predicted profile (Fig. 8).

## Discussion

This study demonstrates the initial application of EUCAST- and CLSI-mandated MH-F media for fastidious organisms in the HFIM, detailing necessary adaptations and successfully achieving long-term culture of *A. pleuropneumoniae* and *P. multocida*. The use of fastidious medium in the HFIM is crucial for simulating intricate microbial and antimicrobial interactions in a controlled environment. The following discussion addresses challenges and considerations linked to integrating fastidious medium into the HFIM.

Fastidious medium, essential for cultivating micro-organisms with intricate growth needs, is valuable in the HFIM for mimicking natural host environments and supporting the growth of challenging-to-culture micro-organisms by providing necessary nutrients and cofactors. While various ‘fastidious media’ exist, the EUCAST recommends MH-F broth for standardization and reproducibility in susceptibility testing and PD studies. However, no previous reports demonstrate MH-F use in perfusing an HFIM system or address associated challenges. Researchers previously used a non-standardized ‘modified fastidious broth’ (mFB) for *N. gonorrhoeae* in the HFIM [14,17], raising concerns about harmonization with EUCAST susceptibility data, critical for accurate PK/PD modelling and dose extrapolation. Perfusion assessment of MH-F in the HFIM revealed challenges such as cellular debris blocking filters, tubing and connections, leading to HFIM failure. Increased cell lysis, related to freeze–thaw cycles, coupled with centrifugation and cell straining, overcame these challenges. However, increased decanting steps raised contamination risks, especially for long runs. Commercially available lysed/laked horse blood, providing reduced contamination risk and increased lysis without requiring cell straining, remains unexplored in this study. Haematin solubilized in sodium hydroxide, an alternative to lysed defibrinated horse blood, may avoid HFIM blockages [12,20]. These modifications offer a practical solution to optimize MH-F, ensuring harmonization with susceptibility testing, maintaining HFIM functionality and enhancing result reliability in studies involving fastidious organisms.

Limited data exist on the growth of fastidious organisms in the HFIM, particularly *A. pleuropneumoniae* and *P. multocida*. This study used the HFIM with ‘improved’ MH-F to assess the growth and maintenance of these organisms for up to 240 h (10 days). The modified fastidious HFIM allowed logarithmic growth immediately after ECS inoculation, reaching a maximal carrying capacity of 10^9^ c.f.u. ml^−1^ for both organisms, and highlighting dynamics during the stationary phase of growth. A comparative growth assay highlighted the importance of MH-F in the HFIM, emphasizing its competence in supporting prolonged cultivation of fastidious organisms. The study revealed distinct temporal dynamics in bacterial population densities of *A. pleuropneumoniae* and *P. multocida* over the 10 day period. However, these results are based on single runs with representative porcine isolates, and a more comprehensive study with multiple wild-type isolates and other fastidious organisms is needed to fully understand the set-up’s limitations.

The requirement for controlled atmospheric CO_2_ for the fastidious organisms in this study is also a requirement for * N. gonorrhoeae* and was included in the studies by Jacobsson *et al*. [16] and VanScoy *et al*. [17]. In this study, it was considered that the inclusion of gas-permeable tubing for the central compartment and gas-vent filters on each of the reservoirs would facilitate gas transfer across the HFIM. Access to CO_2_ may play an important role for capnophiles, such as *N. gonorrhoeae*, and for other fastidious organisms such as those reported in this study. However, the means of gas transfer is not often highlighted when reporting the HFIM. As such, the HFIM previously implemented may not be comparable to our set-up, highlighting the necessity for standardization of the HFIM as described by Sadouki *et al*. [4].

A key feature of the HFIM is its use to emulate dynamic AMD profiles modelling the *in vivo* PK for a given host species. Considering the initial challenges outlined in this study, it was anticipated that MH-F may inhibit or delay equilibration of AMD between the CR and the ECS. The ‘improved’ MH-F and HFIM modifications described were expected to ensure that equilibration could be achieved. To explore the dosing aspect, a florfenicol PK profile in pigs was simulated and showed good agreement between the dose administered and that measured in the ECS. This indicates that for this type of administration rapid equilibration across the fastidious HFIM is achieved, although further studies should explore alternative dose administrations such as long-term infusion and alternative antimicrobial compounds.

In conclusion, the incorporation of fastidious medium in the HFIM represents a crucial advancement in the simulation of complex microbial and antimicrobial interactions in a controlled environment for the purposes of long-term PD assessment. Standardized fastidious media are required to ensure that experimental studies align across laboratories and countries. Further steps should be taken to optimize media clarity to prevent system clogging and maintain gas transfer to optimize bacterial growth. Our study showed that with simple modifications it is possible to maintain the growth of fastidious organisms, specifically *A. pleuropneumoniae* and *P. multocida*, for up to 10 days. Future studies should focus on identifying the limitations of the fastidious HFIM with a range of antimicrobial compounds, dosing profiles and fastidious organisms.

## supplementary material

10.1099/acmi.0.000744.v3 Uncited Table S1.

## Abbreviations

AMD: antimicrobial drug
CAMHB: cation-adjusted Mueller-Hinton broth
CFU: colony forming units
CLSI: Clinical and Laboratory Standards Institute
CR: central reservoir
ECS: extra-capillary space
EMA: European Medicines Agency
EUCAST: European Committee on Antimicrobial Susceptibility Testing
HFIM: hollow-fibre infection model
LOQ: limit-of-quantification
mFB: modified fastidious broth
MH-F: Mueller-Hinton fastidious
PD: pharmacodynamic
PK: pharmacokinetic
RBC: red blood cell
