# Supplementary material for: Hollow-fibre infection model: adaptations for the culture and assessment of fastidious organisms
Source: Access Microbiol. 2024 Jun 28;6(6):000744.v3. doi: 10.1099/acmi.0.000744.v3 (PMC11261730; doi:10.1099/acmi.0.000744.v3)
Supplement: Uncited Table S1. [file acmi-6-00744-s001.pdf]

Supplementary Material

Table S1: Supplier, product number, and description of materials used in this study.

Supplier	Product/Cat. No.	Item Description
Fisher	10569215	BD Plastipak™ Plastic Concentric Luer-Lock Syringe, 20 mL; pk/120
Fisher	15879152	Fisherbrand™ Sterile Syringes for Single Use, 10 mL
VWR	CARD92318	Self Seal Sterilisation Pouches, 330×457 mm; pk100
Glentham Life Science	GV0860	beta-Nicotinamide adenine dinucleotide, 5g
Merck	90922-500G	Mueller Hinton Broth 2
Fisher	15349067	Terumo™ 3-Part 50mL Luer Lock Syringes
Fisher	11597522	Fisherbrand™ Sterile Cell Strainers; 70um
StarLab	E1450-0800	50ml Centrifuge Tube, Conical (Sterile), Racked
Merck	70191-500G	Mueller Hinton Agar suitable for microbiology, NutriSelect® Plus 500g
Fisher	10172852	Gilson™ Pharmed Extension Tubing; 3m; 2mm ID
Fisher	10163572	Gilson™ Pharmed Extension Tubing; 3m; 1mm ID
VWR	MFLX96410-16	L/S® Precision Pump Tubing, Platinum-Cured Silicone, L/S 16; 25 ft
Bio-rad	7318220	Low-Pressure System Fittings Kit
Watson-Marlow	978.0205.00+	Marprene Manifold Tubes 2.05mm Bore Purple/Purple (6/pk)
Watson-Marlow	978.0102.00+	Marprene Manifold Tubing HS 1.02 mm Bore White/White (6/pk)
TCS Bioscience	HB001B	Horse Blood Defibrinated; 1 Litre (bag)
VWR	SCOT292632806	Narrow tubing connection cap with 2-ports, 54×42 mm (Ø×H)
VWR	229-0134	Non return valve with embossed directional arrow
StarLab	N2400-9007	Protection filter with connective tubing, 1 Box × 1 Piece
Covetrus	VPNEE01	Needle Free Valve
Covetrus	VCVAL03	4 Way Stopcock Off Sc4 (W1fg) sng
Glentham Life Science	GV0860	beta-Nicotinamide adenine dinucleotide, 5g
Produlab	G0756	Florfenicol
